# Natural Erosion of Sandstone as Shape Optimisation

**DOI:** 10.1038/s41598-017-17777-1

**Published:** 2017-12-11

**Authors:** Igor Ostanin, Alexander Safonov, Ivan Oseledets

**Affiliations:** 1 Center for Computational and Data-Intensive Science and Engineering, Skolkovo Institute of Science and Technology, Moscow, Russia; 2 Center for Design, Manufacturing and Materials, Skolkovo Institute of Science and Technology, Moscow, Russia

## Abstract

Natural arches, pillars and other exotic sandstone formations have always been attracting attention for their unusual shapes and amazing mechanical balance that leave a strong impression of intelligent design rather than the result of a stochastic process. It has been recently demonstrated that these shapes could have been the result of the negative feedback between stress and erosion that originates in fundamental laws of friction between the rock’s constituent particles. Here we present a deeper analysis of this idea and bridge it with the approaches utilized in shape and topology optimisation. It appears that the processes of natural erosion, driven by stochastic surface forces and Mohr-Coulomb law of dry friction, can be viewed within the framework of local optimisation for minimum elastic strain energy. Our hypothesis is confirmed by numerical simulations of the erosion using the topological-shape optimisation model. Our work contributes to a better understanding of stochastic erosion and feasible landscape formations that could be found on Earth and beyond.

## Introduction

**Figure 1:**
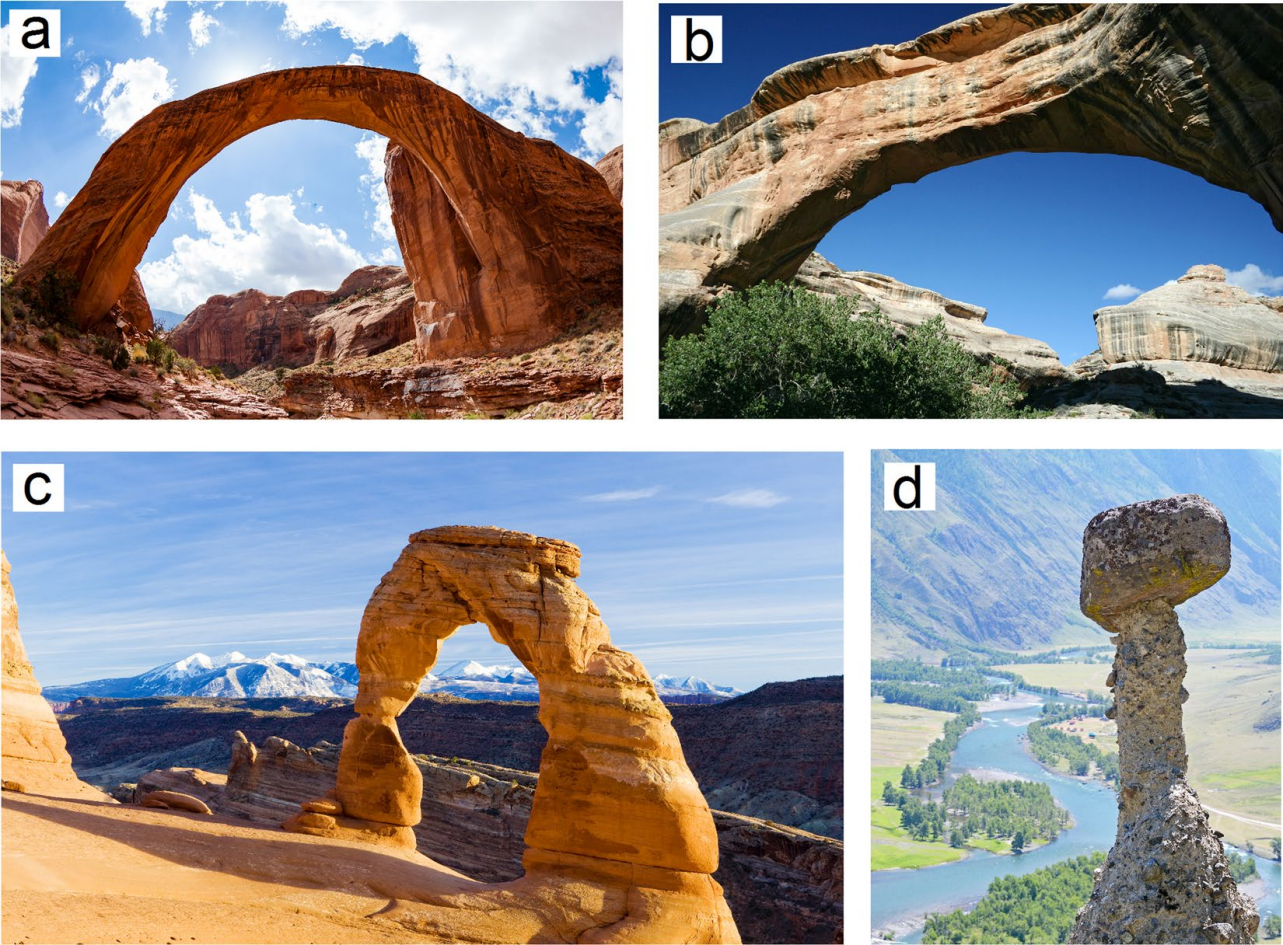
Exotic geological formations. (**a**) Rainbow bridge arch in Utah, USA. (**b**) Sipapu Natural Bridge, Utah, USA. (**c**) Delicate arch in Utah, USA. (**d**) “Stone mushroom” pillar formation in Altai region, Russia. Image source - depositphotos.com. This figure is not covered by the CC BY licence. Image credits: Richard Semik (**a**), David Frederich (**b**), Richard Semik (**c**), Elena Gurdina (**d**). All rights reserved, used with permission.

Unusual sandstone formations, such as natural arches, balanced rocks, natural pillars *etc*. (Fig. 1), have always been a source of amazement and curiosity for many generations of geologists (see^1^ for the detailed overview of the research efforts in this area, since antiquity till the end of the 20-th century). It was always clear that these formations have been sculpted by wind and water, but until recently, the reason for their non-trivial shapes remained a mystery. The first rigorous experimental and theoretical study of the erosion mechanisms behind the formation of natural arches was carried out by Bruthans, J. *et al*.^2^. The cornerstone idea behind this excellent work is the negative feedback between stress and erosion, conditioned by grain interlocking due to geostatic (gravitational) stresses. Using this simple basis, authors provide a qualitative explanation of the mechanisms of formation of exotic geostuctures that is further supported by the experiments and numerical stress analysis. For example, an arch forms when the original structure has two localized support points, causing stress concentrations. Weathering, abrasion and wind deflation removes the granular material in the regions subjected to relatively small compressive stresses, leaving more stressed and consolidated material intact, which, after many loops of erosion, leads to a distinctive natural arch structure.

This idea, undoubtedly fascinating by itself, looks even more interesting in a light of recently emerged engineering techniques of shape and topology optimisation. It appears that one can build an exact correspondence between natural erosion and hard-kill algorithms of shape optimisation. This leads to a number of interesting considerations, which constitute the core of our work. In particular, we demonstrate that the negative feedback between stress and erosion, discovered in^2^, leads to shape optimisation of the rock, locally minimising its elastic strain energy. Below we illustrate this idea with a simple analytical model of erosion and the numerical modelling, based on the approaches, developed in the field of shape and topology optimisation.

## A simplified model of erosion

Natural wind or water erosion is extremely complex multiphysics process, involving elastic deformations, fluid flow, frictional forces, electrochemistry, microcracks, biogenic factors *etc*.^1,3^. However, its most important features can be extracted with elementary considerations. In order to bridge the natural erosion with shape optimisation, we suggest the following simplistic yet predictive model of erosion. Hereafter we will be talking about one particular kind of erosion - wind deflation, which acts uniformly on the whole surface of the eroded rock, unlike abrasion or water erosion that are often highly non-uniform, and cause uneven structures like the one shown in Fig. 1(c). A realistic model of a sandstone erosion with abrasive particles, which has been identified as an important mechanism of wind erosion of a sandstone (*e.g*.^4^), can be easily developed based on similar principles. We limit our consideration to sandstone-like materials that are well described by Mohr-Coulomb failure criterion.

**Figure 2:**
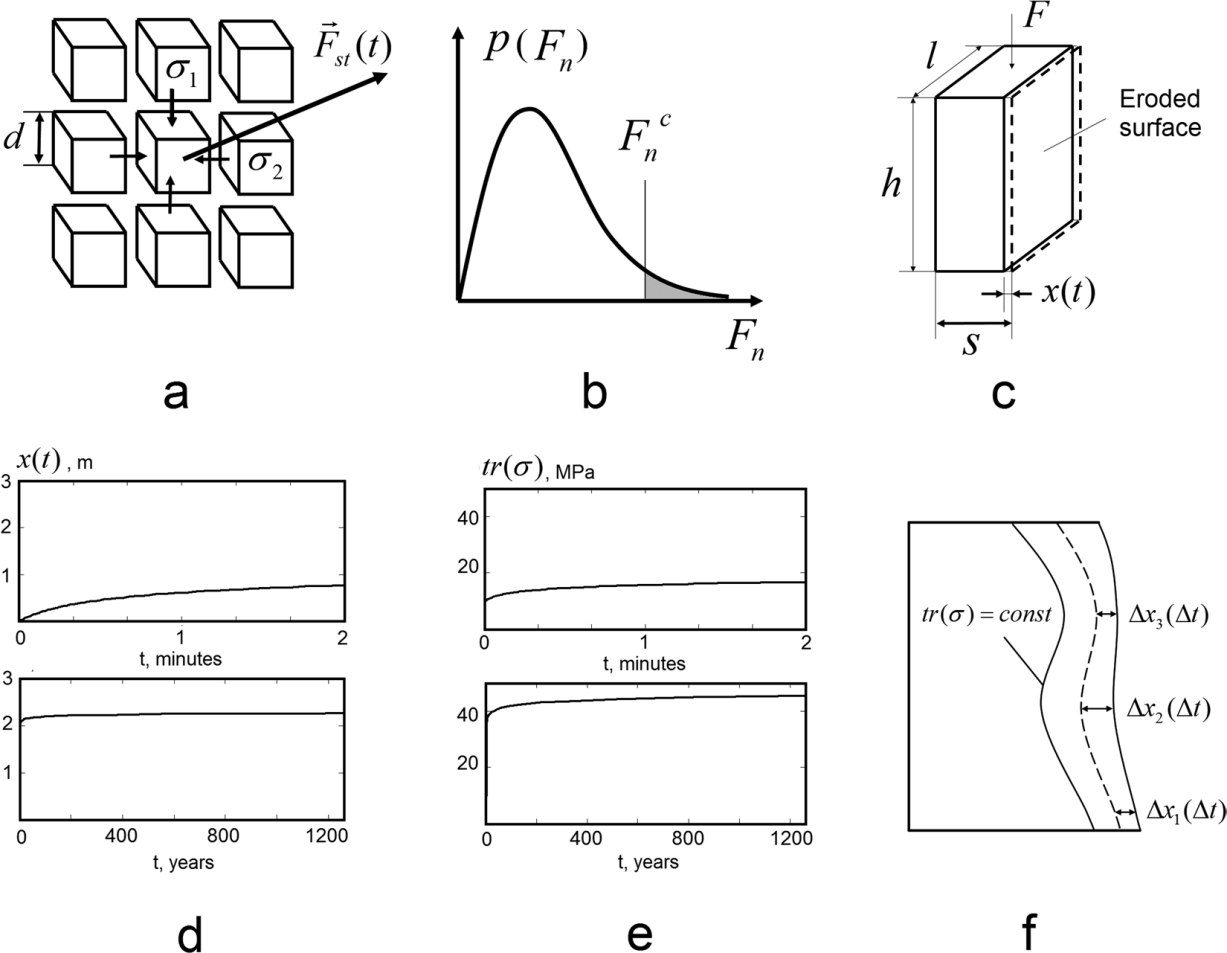
Simplified model of stochastic wind erosion. (**a**) Schematics of a model for the sandstone surface. (**b**) Distribution *p*(*F*_*N*_). (**c**) Model one-dimensional problem of erosion of a rectangular block. (**d**) Position of erosion front and (**e**) trace of a stress tensor as functions of time for two different time scales. (**f**) The case of non-uniform stress (qualitative illustration).

Assume that the volume of the stone is composed of regularly arranged cubic particles (voxels) (Fig. 2(a)). Particles are bound together with cohesive forces that do not depend on local stress state, and frictional forces, that depend linearly on local compressive stress (Mohr-Coulomb law of dry friction). Consider the case when principal compressive stresses are co-oriented with voxel faces (*σ*_3_ = 0 due to traction-free boundary condition on the surface). Assume now that the surface face of each voxel is subjected to a stochastic drag force, changing randomly with time. In order to separate a voxel from the surface, the normal component of the drag force should exceed certain critical value, conditioned by the cohesion between the particle surfaces *c* and the coefficient of dry friction *μ*. Summing up cohesive and frictional contributions from four sides of the voxel, we obtain the following expression for this critical value:

1 $${F}_{n}^{c}(\sigma )=2{d}^{2}\mathrm{(2}c+\mu ({\sigma }_{1}+{\sigma }_{2}))=2{d}^{2}\mathrm{(2}c+\mu tr(\sigma ))$$

It is easy to demonstrate that the last invariant expression holds true for arbitrary orientation of principal stresses *σ*_1_ and *σ*_2_ with respect to the voxel grid. In order to simplify our consideration, we did not take into account different coordinations of the particles, assuming that every surface particle is confined by its 4 nearest neighbours.

The process of a particle separation cannot be instantaneous - it takes certain characteristic time *δt*, during which the normal component of the drag force should exceed the critical force. For simplicity, we assume that this time is constant and does not depend on the local stress and the magnitude of the non-equilibrated normal component of the drag force. Irrespectively of the distribution of the drag force *p*(*F*_*n*_), we can define the average number of particles separated from the surface with the area *S* on the timeline Δ*t* as:

2 $$N(\sigma )=\frac{S}{{d}^{2}}\frac{{\rm{\Delta }}t}{\delta t}\,{\int }_{{F}_{n}^{c}(\sigma )}^{\infty }\,p({F}_{n})d{F}_{n}$$

Relationships 1 and 2 establish the negative feedback between stress and erosion, pointed out in^2^. The distribution for the stochastic drag force is unknown^5^; however, we can develop a simple model of erosion using two reasonable assumptions. First - we assume that the distribution of drag force is the same at every point of the rock’s surface (this can be accepted for the case of uniform wind deflation). Second - particle detachment from the surface of the rock corresponds to the tail of the distribution *p*(*F*_*n*_), *i.e*. it is a relatively rare event (Fig. 2(b)).

In order to illustrate the basic features of uniform erosion defined by (1), (2), consider simple one-dimensional example - the erosion of a rectangular block under uniaxial compression (Fig. 2(c)). The block is eroded along one of its faces. The uniform compression with vertical force *F* causes a compression stress *F*/*l*(*s* − *x*(*t*)), increasing with the propagation of erosion front *x*(*t*). For the purpose of illustration, let us assume the exponential decay of the drag force distribution (one can show that any other distribution that are often used to describe wind variations (Gaussian, Raleigh or Weibull^5^) would yield qualitatively similar results):

3 $$p({F}_{n})=C\cdot {e}^{(-{F}_{n}/{F}_{n}^{0})}.$$

Then the equation (2) straightforwardly leads to the following ordinary differential equation for the erosion process:

4 $$\frac{dx}{dt}=C^{\prime} \cdot {e}^{(-{C}^{^{\prime\prime} }\frac{F}{l\cdot (s-x)})}$$

The derivation of this equation and its analytical solution is presented in the supplementary material. Figure 2(d,e) give an illustration of this solution in terms of the position of the erosion front *x* and trace of the stress tensor *tr*(*σ*) as a function of time, for two different time scales. One can observe that as long as the principal stress *σ*_1_ is growing due to shrinkage of the cross-section *S* = *l* · (*s* − *x*(*t*)), the propagation of the erosion front slows down rapidly. In our model problem the block that is initially quickly eroded on a time scale of minutes, becomes then stable on geological time scales. These observations lead us to two important conclusions:

(i) If erosion facilitates stress concentration (which is the case for free-standing structures), the erosion process slows down with time, reaching at some point a very stable shape, almost unaffected by further erosion. This model property fully agrees with conclusions made in^2^.
(ii) due to the strong dependence of erosion rate on the local stress, in the case of non-uniform local stress distribution, the eroded rock will tend to attain a shape of the local level surface of the function of the kind:

5 $$tr(\sigma )=const$$

Figure 2(f) gives a qualitative illustration of this effect: relatively small change in local stress causes the dramatic change in the erosion rate, which leads to shaping the eroded material along level lines of (5). One should note, however, that, as in any other hard-kill optimisation, (5) may not hold globally in more complex configurations, where material removal at one surface point can lead to stress concentration increase in another. The level of stress reached in stable configurations depends on a number of problem parameters - geometry of the problem, magnitudes of surface and volume loads, cohesion, friction angle and intensity of erosion (see supplementary material for the parameters of the model example showcased in Fig. 2).

In other words, our simple model leads us to a conclusion that natural erosion works similarly to a *hard-kill* method of shape optimisation (see, *e.g*.^6–9^). Within such shape optimisation techniques, we seek for the shape and topology of a domain that, given a certain set of constraints, minimizes the cost functional, *e.g*. strain energy stored by the elastic domain. In such procedures, optimisation is carried out by successive material removal according to a certain sensitivity function. This sensitivity function can be based on either a rigorous derivation (topological derivatives^9^) or to be purely heuristic (*e.g*. von Mises stress in ESO^6^). Such sensitivity function in our case is defined by the physics of the erosion process and is nothing but a trace (first invariant) of a stress tensor. This sensitivity does not precisely coincide with any sensitivities associated with the cost functionals commonly studied in shape optimisation. However, our numerical experiments showcased below indicate that the trace of a stress tensor can serve as a successful heuristic criterion for the local minimisation of the elastic strain energy.

The reason for that is simple. Rigorous algorithms of hard-kill energy minimisation are based on the successive material removal along the level lines of a topological derivative^9^ - the sensitivity of the strain energy functional to an introduction of a spherical cavity at a point *x* inside the domain. In presence of a constant body force the analytical expression for topological derivative has the following form (*d* = 2, 3 for 2D and 3D cases correspondingly):

6 $${D}^{T}(x)={C}_{1}+{C}_{2}\,\sum _{d}\,{\sigma }_{i}^{2}+{C}_{3}{(\sum _{d}{\sigma }_{i})}^{2}$$

Consider first 2D case. If the hard-kill algorithm removes material only near the traction free surface of the material (${\sigma }_{2}\ll {\sigma }_{1}$), then the level lines of a topological derivative are described with a simple equation

7 $${\sigma }_{1}(x)=const,$$

which is equivalent both to level lines of von Mises stress $(\sqrt{{\sigma }_{1}^{2}+{\sigma }_{2}^{2}-{\sigma }_{1}{\sigma }_{2}})$ and stress trace (*σ*_1_ + *σ*_2_) under the same condition. Therefore, in case of two-dimensional surface erosion problems like the formation of slender arches the stress trace coincides with the exact analytical criteria for the material removal in a hard-kill optimisation algorithm.

In general 3D case we have two dominant principal stresses near traction free surface (*σ*_1_, ${\sigma }_{2}\gg {\sigma }_{3}$), and therefore, similar equivalence between different material removal criteria does require an additional condition (${\sigma }_{2}\ll {\sigma }_{1}$) to be met. This is the case for a wide class of structures, including *i.e*. most of the 3D structures considered in our work. Therefore, the erosion processes are capable to optimise shapes in both 2D and 3D setting.

At this point we can capitalize the major point of our work - *stochastic wind erosion (deflation) works as shape optimisation, locally minimising the elastic strain energy stored in a free-standing natural structure*.

It is important to capitalize that both hard-kill methods of energy minimisation and our erosion model are fundamentally unable to provide *global energy minimisation*, *i.e*. finding a unique and globally optimal shape minimizing the elastic energy. This is due to the obvious constraints imposed by unidirectional material removal, and (in case of erosion) material elimination only on the surface of the domain. Clearly, such optimisation procedures prohibit vast classes of the optimal solutions that could be reached by adding material or nucleating cavities within the domain. Therefore, in our work we talk only about local minimisation of the elastic strain energy.

The simple model described above can serve as a basis for the predictive numerical modelling of the erosion. Although direct Monte-Carlo numerical simulation of a stochastic destruction is possible, it is limited to a relatively small number of constituent particles and timesteps, and therefore can not resolve realistic length and time scales. Instead, we opt for deterministic modelling approach based on the developments in the field of shape optimisation. We build our model of erosion based on the following simplification: we assume that the erosion rate is constant, however, there exists a critical value of the stress trace *σ*_*c*_, after which the erosion stops completely (such assumption agrees with the interpretation suggested in^2^). As a baseline, we utilize a combination of SIMP(Solid Isotropic Material with Penalization) scheme of stiffness interpolation^10^ and the hard-kill strategy of material removal. The details pertaining to our modelling technique are discussed in the Methods section. Below we discuss a set of examples that demonstrate that this relatively simple numerical modelling technique captures all the important features of uniform erosion of a sandstone.

## Numerical simulations

Here we consider few illustrative examples that demonstrate the validity of our ideas and predictive power of our simple numerical model of erosion. All the examples utilized the following mechanical properties of a sandstone: *E* = 10 *GPa*, *ν* = 0.3, *D* = 2000 *kg*/*m* ^3^. Critical stress after which the erosion rate was considered negligible was accepted to be *σ*_*c*_ = 30 kPa.

**Figure 3:**
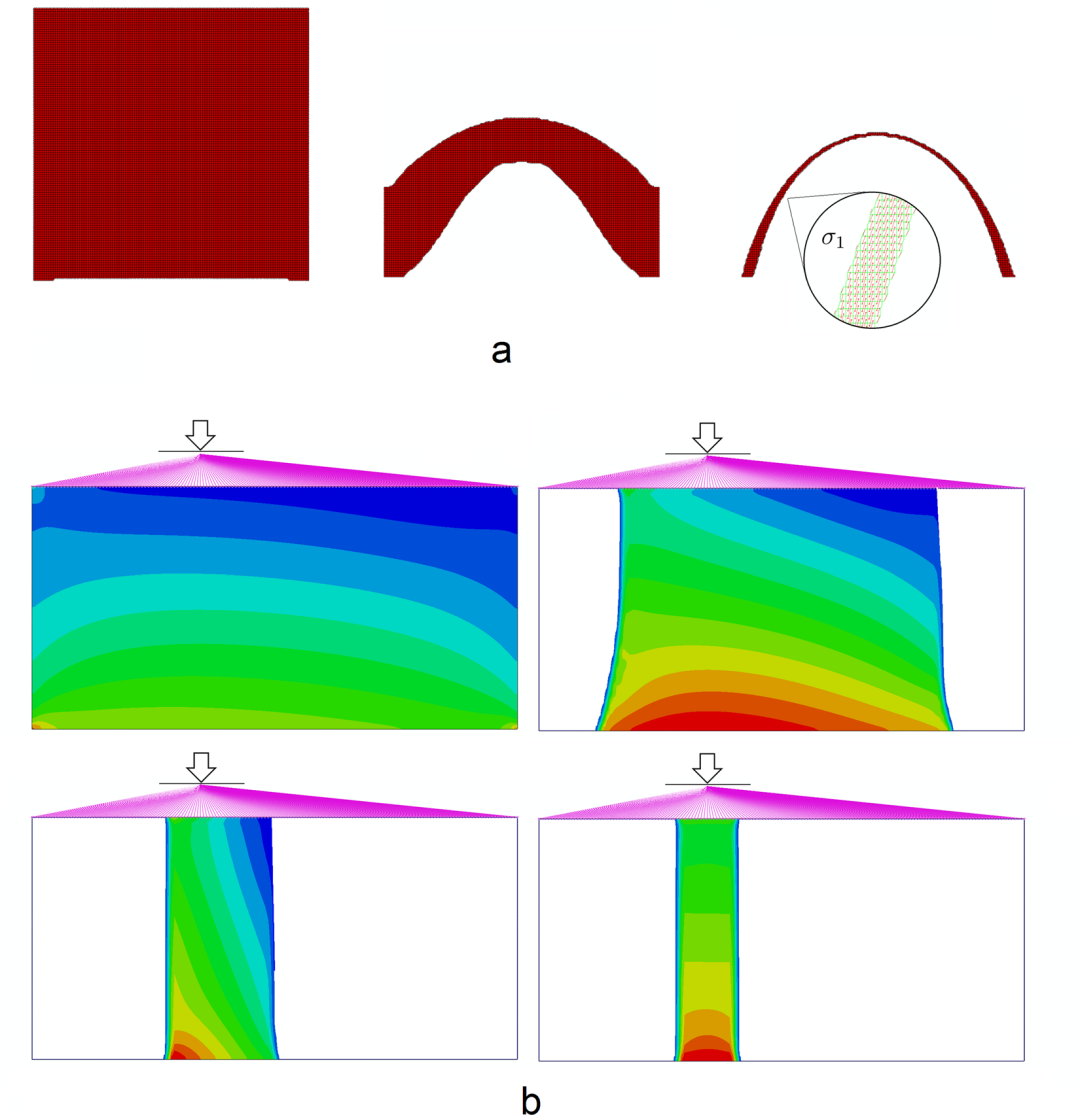
Two-dimensional modelling of erosion. (**a**) Arch with two points of support. An inset on the third snapshot demonstrates the principal stress distribution achieved in the equilibrated arch shape. (**b**) Self-balancing of the pillar under off-axial load.

Consider first a two-dimensional example demonstrating the formation of arch shape by an erosion process. The initial domain is composed of 150 × 150 × 1 regular grid of cubic finite elements (15 × 15 × 0.1 m). The only loading in the system is the gravitational body force. The erosion is initialized at every surface element excluding 30 elements forming two separated support spots at the base of the square block. Figure 3(a) gives the evolution of the eroded shape with time (see also video 1 in the supplementary information).

The resulting arch strongly resembles the famous “Gateway Arch” in St. Louis, USA. Despite common misconception, the shape of that arch is not a catenary, but a “flattened catenary” – subclass of a “weighted catenary” (see the extensive discussion in^11,12^) - the line formed by a hanging chain with non-uniform weight distribution. The analysis of displacements and principal stress components in the structure, generated by our optimisation procedure, indicates that resulting structure belongs to a class of “weighted catenary” curves, *i.e*. the only principal stress at its every cross-section is co-aligned with the median line of the structure (Fig. 3(a)). Such structures are known to be locally optimal in terms of minimum strain energy, since any deviation from the weighted catenary will result in bending of the structure, inducing significant increase in a stored strain energy. Therefore, we can clearly see that our optimisation procedure, based on the physically just model of erosion, creates structures locally minimising their elastic strain energy.

The second important example illustrates how our simple erosion mechanism leads to self-balancing of massive structures, like ones shown in Fig. 1(c,d). The original domain consists of 150 × 75 × 1 elements (15 × 7.5 × 0.1 m). Elements along the bottom of the block are rigidly fixed. It is loaded by a gravitational body force, and by vertical off-center concentrated force, which, using direct kinematic links, is transferred to all upper surface nodes of the block. This system of kinematic links models a massive body resting on the eroded foundation. The erosion starts at the two side surfaces of the block. Figure 3(b) gives four consequent snapshots of the shape of the foundation with added maps of a trace of the stress tensor (see also video 2 in the supplementary information). We can see that the erosion process initiates with the same rate on left and right side. However, initial asymmetry of the loading causes stress concentration at the left side, which slows down the erosion. The erosion on the right side develops with an unchanged rate, until the asymmetry of the loading is alleviated. The erosion process results in a pillar that is ideally centered with the axis of the applied force. This example illustrates how the dependence of the erosion on the trace of the stress tensor creates a negative feedback loop, stabilizing a massive geological formation.

**Figure 4:**
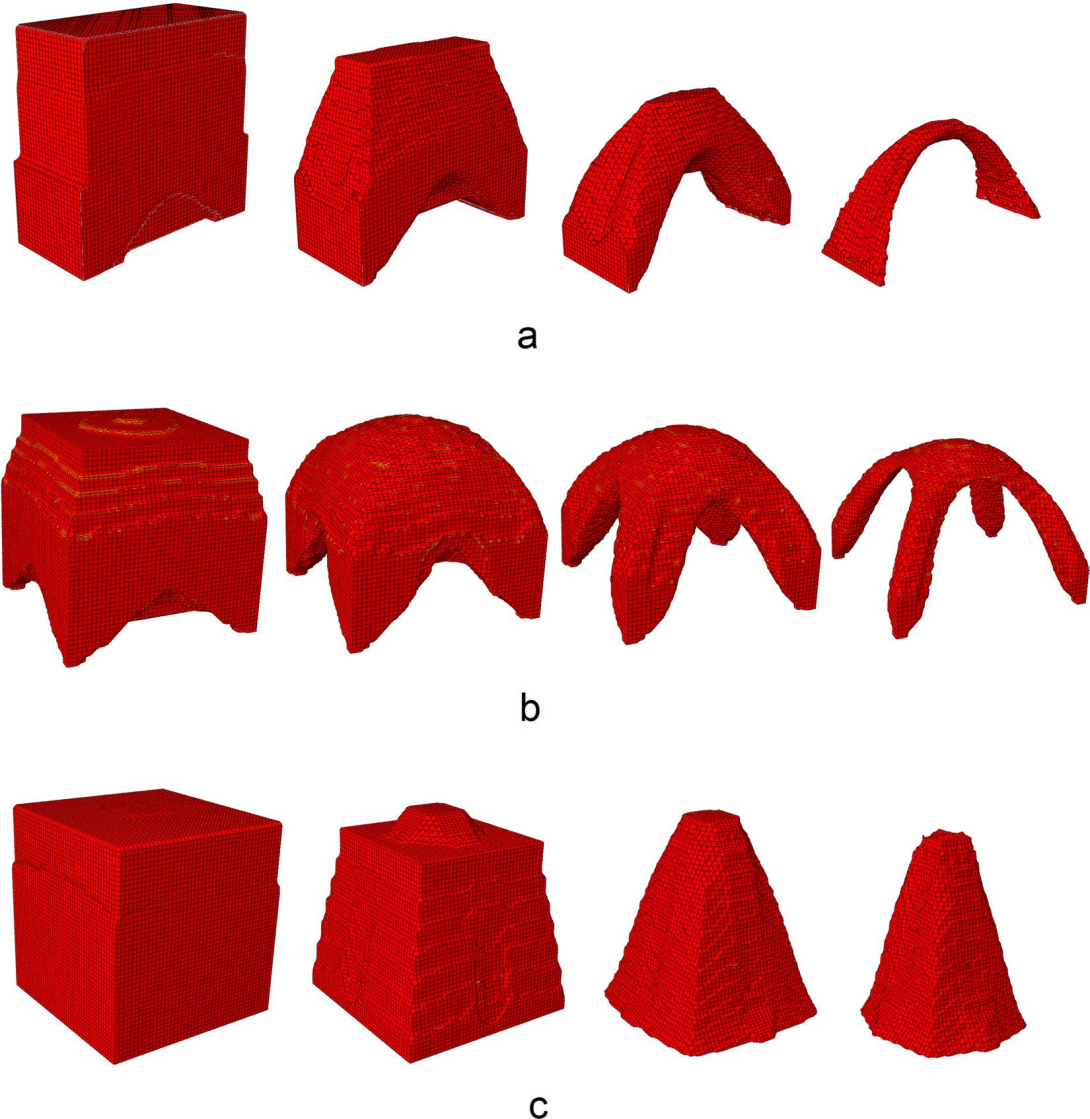
Three-dimensional modelling of erosion. (**a**) Arch with two points of support. (**b**) Arch with four points of support. (**c**) “Stone mushroom” pillar.

Next three examples demonstrate the application of our algorithm to more realistic three-dimensional structures. The first one is the realistic 3D model of a natural arch, similar to ones presented in Fig. 1(a,b). We start with a rectangular block of material consisting of 50 × 50 × 20 elements (5 × 5 × 2 m). We define two support spots (2 × 20 elements) along bottom edges of the domain. Figure 4(a) and video 3 in the supplementary information gives the time evolution of the eroded shape. One can see that the erosion resulted in a three-dimensional weighted catenary arch with a complex cross-section, similar to ones found in nature. Figure 4(b) gives the time evolution of the cubic eroded block (50 × 50 × 50 elements, 5 × 5 × 5 m)) with four spots of support (5 × 5 elements each). Although the resulting structure does not remind any geological formation on Earth or beyond, it is just as physically feasible as the other considered examples. The last benchmark is the model of a “stone mushroom” depicted in Fig. 1(d). It is modeled as a cubic eroded foundation (50 × 50 × 50 elements, 5 × 5 × 5 m)) loaded with the gravitational body force and the additional concentrated traction acting on a spot in the center of the upper side of the cube (10 × 10 elements) imitating the piece of rock that is not subjected to erosion. The resulting conical structure reproduces well the existing geological formations.

Sizes of the structures observed in the simulation agree well with the sizes observed in nature. We note that the lower bound on admissible structure size is roughly defined by the length scale *l* = *σ*_*c*_/*ρg*, defining spatial scale where the gravity-induced stresses are comparable with the strength of material. In our case *l* = 1.5 m, meaning that arch structures can not be observed at smaller scales. For more accurate discussion on spatial scalings of catenary arch structures the reader is referred to^11,12^.

## Discussion

Here we discuss the main findings pointed out above as well as their immediate implications. First, under certain conditions, differential laws of natural erosion, that originate in the fundamental law of dry friction, lead to optimal shapes that locally minimise the elastic strain energy stored in the rock bulk. This amazing property is nothing but a manifestation of the fundamental physical principle of minimum potential energy. It states that a continuous or discontinuous system shall transit to a state that minimizes the total potential energy with leftowers of the energy converted to kinetic energy (heat). The key feature of sandstone erosion that enables explicit energy minimisation is an interplay between continuous and discontinuous behaviors. Sufficient contact between sandstone particles leads to continuous elastic fields induced in a compressed sandstone. These fields serve as a mechanism that links local isolated events of particle detachments for the sandstone surface into a continuous mechanism of local minimisation of the potential energy.

Second interesting observation is that this natural mechanism of energy minimisation almost precisely coincides with so-called “hard-kill” optimisation algorithm of topology optimisation^6–8^. Strictly speaking, natural erosion can be viewed as a Monte Carlo variant of a “hard-kill” local energy minimisation. The possibility of physical realisation of a mathematical optimisation algorithm raises an interesting question of finding other physical processes that can be harnessed for industrial production of optimal structures (particularly, at a nanoscale).

The third important outcome of our work is a fully functional code for realistic numerical modelling of the erosion of a sandstone and similar materials. Our numerical modelling completely affirmed the earlier suggested hypothesis that the formation and self-balancing of massive geostructures is conditioned by the negative feedback between stress and erosion. Therefore, our results provide the researchers with quantitative instruments for realistic modelling of physically admissible eroded shapes. This can be a helpful instrument, for example, for the analysis of geostructures found on Mars, where eroded sandstone structures are ubiquitous.

## Concluding remarks

In this work, we tried to develop an exciting idea of a negative feedback between stress and erosion, present in sandstone-like materials, offered in^2^. This has resulted in a number of interesting findings. We have shown that under a set of physically meaningful conditions natural erosion works as a hard-kill method of shape optimisation. It was demonstrated that the criterion linking stress and erosion, which originates from the fundamental laws of dry friction, leads us to a conclusion that erosion leads to local minimisation of the elastic strain energy of the resulting structure. We have shown that in spite of the stochastic nature the results of the erosion process can be modeled with deterministic shape optimisation approaches. We conclude that the local energy minimisation is nothing but a manifestation of principle of minimum potential energy. Our results provide the researchers with quantitative instruments for realistic modelling of physically admissible eroded shapes.

## Methods

### Numerical modelling of natural erosion

As a baseline of our numerical modelling technique, we utilize SIMP (Solid Isotropic Material with Penalization) scheme of stiffness interpolation in the discretized domain^10,13^. At every iteration of the optimisation process, the problem is represented as a regular two- or three-dimensional mesh of finite elements with varying densities. In our case, we allow for variation of densities only in the vicinity of the surface, which is defined as an interface between elements with zero and nonzero densities. Such formulation imitates the surface erosion, that can cause only a limited class of topology changes, and reliably excludes numerical artifacts inherent to “hard-kill” optimisation methods^14^.

The model utilizes finite element method (FEM) modelling of the elastic behavior of a sandstone. Every *i*-th finite element of an initial domain has the associated density *ρ*_*i*_ ∈ [0, 1]; at the initial moment of the simulation all the *ρ*_*i*_ = 1. Gravitational body force with the magnitude *g* · *D* · *ρ*_*i*_ is applied to every finite element (*D* is specific gravity of the sandstone).

The elastic properties of *i*-th finite element are described in terms of its Young’s modulus *E*_*i*_ and Poisson’s ratio *ν*_*i*_. The latter does not depend on the element density; a relationship between Young’s modulus of *i*-th finite element *E*_*i*_ and its density *ρ*_*i*_ is given by:

8 $${E}_{i}={E}_{{\rm{\min }}}+({E}_{{\rm{\max }}}-{E}_{{\rm{\min }}}){\rho }_{i}^{p},$$

where *E*_max_, *E*_min_ are minimum and maximum admissible Young’s moduli, *p* is the parameter of penalization. Maximum modulus *E*_max_ corresponds to actual modulus of a sandstone, *E*_min_ is taken to be very small, but nonzero, in order to avoid singularities when manipulating with FEM stiffness matrices.

The erosion of the material occurs only in the elements in the vicinity of the surface. Within our approach, an element is considered to be a surface element if it has nonzero density and at least one of its neighbours has zero density. Two elements are considered to be neighbours if the distance between their centers does not exceed *R*_*c*_, which corresponds to few sizes of a finite element.

The density distribution *ρ*_*i*_ at *n* + 1-th time step for *i*-th element is defined as follows:

9 $${\rho }_{i}^{n+1}=\{\begin{array}{llll}{\rho }_{i}^{n}-max({\rho }_{i}^{n},\theta ) & {\rm{if}}\quad tr(\sigma )-{\sigma }_{c}\ge 0 & {\rm{AND}} & i\in {\rm{\Gamma }},\\ {\rho }_{i}^{n} & {\rm{if}}\quad tr(\sigma )-{\sigma }_{c} < 0 & {\rm{OR}} & i\notin {\rm{\Gamma }},\end{array}$$

where *θ* ∈ [0, 1], *σ*_*c*_ - critical value of the stress trace function.

It is known that the discretizations with intermediate densities often lead to certain numerical difficulties, such as checkerboard instabilities and mesh dependency of the solution^14^. In order to avoid these, we use the approach based on density filtering: when computing mechanical characteristics according to (8), the density *ρ* is replaced with the filtered density $\bar{\rho }$. Filtering operation performs weighted averaging over neighboring elements, as described in^15^.

The algorithm described above is implemented in ABAQUS^16^ using the modification of the structural topology optimisation plug-in, UOPTI, developed previously^17^.

## Electronic supplementary material

Supplementary Information

Movie 1.

Movie 2.

Movie 3.

Movie 4.

Movie 5.
