# Author Correction: Natural Erosion of Sandstone as Shape Optimisation

**DOI:** 10.1038/s41598-018-24100-z

**Published:** 2018-04-13

**Authors:** Igor Ostanin, Alexander Safonov, Ivan Oseledets

**Affiliations:** 1 Center for Computational and Data-Intensive Science and Engineering, Skolkovo Institute of Science and Technology, Moscow, Russia; 2 Center for Design, Manufacturing and Materials, Skolkovo Institute of Science and Technology, Moscow, Russia

Correction to: *Scientific Reports* 10.1038/s41598-017-17777-1, published online 11 Decemeber 2017

This Article contains typographical errors in the Acknowledgements section.

“Authors gratefully acknowledge the financial support from Russian National Foundation under the grant 15-11-00033.”

should read:

“Authors gratefully acknowledge the financial support from Russian Science Foundation under the grant 15-11-00033.”

