# Supplementary material for: Natural Erosion of Sandstone as Shape Optimisation
Source: Sci Rep. 2017 Dec 11;7:17301. doi: 10.1038/s41598-017-17777-1 (PMC5725602; doi:10.1038/s41598-017-17777-1)
Supplement: Supplementary file 1 — Supplementary Information [file 41598_2017_17777_MOESM1_ESM.pdf]

Natural Erosion of Sandstone as Shape Optimisation

Igor Ostanin, Alexander Safonov, & Ivan Oseledets

Supplementary material

One-dimensional analytical model of erosion

Here we give a solution of the ordinary differential equation of one-dimensional erosion of a rectangular block, stressed as depicted in Fig. 3(A) (The case of biaxial compression of the block is completely analogous). According to the equation (2) in the paper, we can write down the expression for a number of particles N leaving the side surface of the block during the time span $\triangle t$ as:

$$N = \frac{hl}{d^2}\frac{\triangle t}{\delta t}\int\limits_{F_n^c}^{\infty} p(F_n)dF_n. \tag{1}$$

Correspondingly, the change in the averaged position of an erosion front in that time span would be given by

$$\triangle x = d\frac{\triangle t}{\delta t}\int\limits_{F_n^c}^{\infty} p(F_n)dF_n. \tag{2}$$

For the sake of demonstration, let us assume that the tail of the distribution $p(F_n)$ is described

by the exponential law (equation (3) in the paper). Then the expression (2) can be re-written as follows:

$$\triangle x = d\frac{\triangle t}{\delta t}\int\limits_{F_n^c}^{\infty} Ce^{-F_n/F_n^0}dF_n = -d\frac{\triangle t}{\delta t}F_n^0 Ce^{-F_n/F_n^0}\mid_{F_n^c}^{\infty} = d\frac{\triangle t}{\delta t}F_n^0 Ce^{-F_n^c/F_n^0}. \tag{3}$$

Recalling that

$$F_n^c = 2d^2(2c+\mu tr(\sigma)) = 2d^2(2c+\mu\sigma_1) = 2d^2\left(2c+\mu\frac{F}{l(s-x)}\right), \tag{4}$$

and taking the limit $\triangle t \to 0$, we obtain the following ordinary differential equation:

$$\frac{dx}{dt} = C' e^{-C''\frac{F}{l(s-x)}}, \tag{5}$$

where $C' = \frac{CdF_n^0}{\delta t}e^{-\frac{4d^2c}{F_n^0}}$, $C'' = \frac{\mu}{F_n^0}$. This equation is straightforwardly integrated by the separation of variables:

$$C'\int dt = \int \frac{dx}{e^{-C''\frac{F}{l(s-x)}}} = -\frac{C''F}{l}\int e^{\frac{C''F}{l(s-x)}} d\left(\frac{l(s-x)}{C''F}\right) = -\frac{C''F}{l}\int e^{1/\xi(x)}d\xi(x), \tag{6}$$

where $\xi(x) = \frac{l(s-x)}{C''F}$. The last integral is evaluated as

$$\int e^{1/\xi} d\xi = \xi e^{1/\xi} - Ei(1/\xi), \tag{7}$$

where $Ei(x)$ stands for Euler integral exponent function given by $Ei(x) = -\int_{-x}^{\infty} \frac{e^{-t}dt}{t}$. The solution of our ODE is therefore given as:

$$x(t):\ t(x) = -\frac{C''F}{C'l}\left(\xi(x)e^{1/\xi(x)} - Ei(1/\xi(x))\right) + C*. \tag{8}$$

enforcing $t(0) = 0$, we obtain the integration constant as

$$C^* = \frac{C''F}{C'l}\left(\xi(0)e^{1/\xi(0)} - Ei(1/\xi(0))\right). \tag{9}$$

The following set of model parameters was used to compute the dependencies presented in Fig. 3(B,C): $h = 5$ m, $l = 2$ m, $s = 3$ m, $d = 1$ mm, $\delta t = 1$ s, $\mu = 0.3$, $c = 1$ MPa, $F_n^c = 400$ kN, $C = 1$, $F = 60$ MN.

Video materials

The following video files are attached:

Movie 1. Two-dimensional model of the arch formation by uniform erosion

Movie 2. Self-balancing of the pillar under off-axial load

Movie 3. Three-dimensional modelling of erosion. Arch with two points of support

Movie 4. Three-dimensional modelling of erosion. Arch with four points of support

Movie 5. Three-dimensional modelling of erosion. ”Stone mushroom” pillar
